# Simplifying data and code sharing

**DOI:** 10.1038/s41467-024-50517-4

**Published:** 2024-07-29

**Keywords:** Research data

## Abstract

Data is the bedrock of scientific progress. At *Nature Communications* we have been making it easier for authors to share their source data by integrating both data and code sharing capabilities into our manuscript tracking system.

As scientific research becomes increasingly data driven, it is important that this data and code is made available to all. Sharing allows other researchers to analyze and build on existing studies, and reuse existing tools, thus accelerating the pace of research. It helps to promote transparency and accountability in scientific research, allowing for more rigorous scrutiny and evaluation of research findings – especially important given reports of a reproducibility crisis in many fields. It can also help the authors, by increasing community engagement with your work and potentially increasing citations. Last, but not least, data sharing is mandated by many funders and institutes, and the number and strength of such mandates will only increase with time.

“To make sharing easier, we have recently made two changes: integrated data deposition with figshare, and integrated code deposition with Code Ocean.”

To make sharing easier, we have recently made two changes: integrated data deposition with figshare, and integrated code deposition with Code Ocean.

The integration of figshare deposition with our manuscript tracking system allows our authors to easily upload a range of data types during the submission process.This data is then available to reviewers throughout the review process, lending greater weight to the claims of the paper. Just as importantly, once the manuscript is published the data will be published, with a permanent DOI, alongside it.

As a generalist repository, figshare is a suitable host for most types of data. However, there are cases where deposition in a specialized database is better suited, or even mandated – in these cases authors should continue to use the most appropriate community repository.

Complementing the generalist approach of figshare, our partnership with Code Ocean allows authors to share code both during review and after publication. If choosing to use Code Ocean integration during the submission of a manuscript, authors will be able to upload their code to be stored privately in the Code Ocean repository. The platform will then generate the corresponding computational environment, including the necessary dependencies, resulting in a self-contained, cloud-based executable capsule to be shared anonymously with the reviewers.This reduces the burden on the reviewers since the installation of code and dependencies is supplied by Code Ocean. Upon acceptance of a manuscript, the final version of the capsule will be published with a permanent DOI. This provides a static reference to the exact software used to generate the results of the publication, supporting reproducibility and building upon efforts from the whole community.

We recognize that data and code sharing is an ever more important component of scientific research, and we are committed to making it easier for authors, reviewers, and readers to access and use supporting data, be it experimental or computational in nature. We look forward to seeing the impacts of these features on scientific research, collaboration, and reproducibility.

